# Mastodon footprints found to be water erosion in the Quebrada de Chalán (Licto, Ecuador)

**DOI:** 10.12688/f1000research.123579.1

**Published:** 2022-11-01

**Authors:** Benito Mendoza, Mauro Jiménez, Pedro Pedro Carretero, Jhonnatan Hernández, Jennifer Loaiza, Daniela Brito, Geonatan Peñafiel

**Affiliations:** 1 Facultad de Ingeniería, Universidad Nacional de Chimborazo, Riobamba, Chimborazo, 060150, Ecuador; 2 Earth Sciences Energy and Environment, YACHAY, Urcuquí, Imbabura, 100650, Ecuador; 3 BMTLAB Laboratories and Engineering, Riobamba, Chimborazo, 060150, Ecuador

**Keywords:** Andean geology, mastodon footprints, water erosion, Chalán

## Abstract

The Chalan ravine is a deep bed creek that runs through Licto (Ecuador). It has been known since the 19th century for the abundance of paleontological remains of Pleiostocene fauna and megafauna in its profiles, where entire remains of mastodons were recovered. The abundance of these remains made one of the high areas, where marmites exist in different forms, was traditionally considered as mastodon footprints. Archaeological prospecting, geographic information system (GIS) technology, unmanned aerial vehicle (UAV), photogrammetry, and the geological study of the place, allowed us to determine that the mythical traces of mastodon were marmites made by the water erosion produced in the same ravine over time.

## Introduction

In the late 19th century, foreign researchers began to arrive in the Quebrada de Chalán, attracted by the legend of the existence of bones of giant hominids. It was Juan Félix Proaño who, in 1884, after a great collapse in the Quebrada, proceeded to excavate the remains of a complete mastodon that had been exposed. These remains were sent to the Central University of Ecuador (Quito), where they disappeared after a large fire ( Branco, 1938; Román, 2010).

Later, other scholars such as Spillmann (1931) and Hoffstetter (1952) came, attracted by the wealth of remains of Pleistocene animals, with the desire to carry out their research.

Therefore, the region is one of the main Pleistocene sites of Ecuador in terms of fossil remains from that period. From then on, new legends emerged with the arrival of these scholars in the first half of the 20th century, one of them being the object of our study, that the area of the marmites were actually mastodon footprints that had been fossilized as they fled before the eruption of the nearby Tulabug volcano. So much so that this legend has been taken as truth, since it was “endorsed by foreign scientists” and, although it had never been reported into a scientific study, it has been transmitted among the inhabitants of the place up to this day. Thus, even today all the living in the area think that the marmites correspond to the aforementioned fossilized traces of mastodons, so that information panels and a marketing campaign have been created in the area. Newspapers such as “La Prensa de Riobamba”, “Diario de Riobamba” or “El Telégrafo” (national) continue to consider these features as traces of mastodons and, from time to time, they take out in their pages reports on the aforementioned footprints, thus feeding the myth and confusing both locals and visitors ( La Prensa, 2021; de Riobamba, 2019; El Telegrafo, 2016).

The objective of this research was to determine whether the existing marks in the study area were the product of (as tradition says) traces of mastodons that lived in the area during the Pleistocene, or were caused by water erosion (marmites) of the rock resulting from the passage of water.

## Methods

Visual archaeological surface survey was carried out in the study area, in which it was determined that there are no archaeological or paleontological remains in the marmite area ( Fernández, 1989). For this purpose, aerial photogrammetry was used using an unmanned aerial vehicle (drone) that includes a camera, obtaining qualitative and quantitative information from the earth’s surface, through a process of recording, measuring, and interpreting photographic images. The UAV used to obtain aerial digital cartography was a DJI Phantom 4 Pro V2.0 multirotor drone with a 20 MP aero-ported camera. The generation of photogrammetric products was carried out in three stages ( Casella, Drechsel, Winter, Benninghoff, and Rovere, 2020; Gasparini, Moreno-Escribano, and Monterroso-Checa, 2020; Stott, Williams, & Hoey, 2020):

‐ In the first stage, the flight path or flight plan was defined using Pix4DCapture software; parameters such as flight area in hectares, flight time in minutes, number of images to be captured, flight height in meters was configured, Pixel size in cm/px, horizontal and longitudinal overlap 75% recommended by the software, speed in m/s, a camera angle of 90°and number of batteries to be used.
‐ The second stage consisted of preliminary survey of the terrain, location of control points (GCP) for correct orthophoto georeferencing and desired elevation models.
‐ In the third stage the flight was carried out on 15 July 2021 at an altitude of 150 m, for 18 minutes, using two batteries, and recording 199 aerial images with a spatial resolution of 4.5 cm/px.

The captured photographs were stored in the drone’s internal memory and downloaded to the computer. The images were processed in the Pix4DMapper software, according to the methodology recommended by the manufacturer and described below:

‐ When the software was started, the new project option was selected and the path where the postprocess files were created was defined.
‐ Once the new project was generated, the photographs captured by the drone were added, the software automatically detects the camera used and the coordinate system; in this case they are geographical coordinates. These coordinates are then transformed into the same software to Mercator’s Universal Transversal UTM.
‐ For 3D processing, 3D Maps processing was selected to obtain orthomosaic, point cloud and digital elevation models, in the initial processing the image scale and geometrically verified pairing were determined.
‐ The processing of the dense point cloud was performed with a classification to improve the generation of the digital MDT terrain model, generate the digital surface model, orthomosaic. Additionally, a process of ortho rectification and contours was performed with a range of 5m.

In addition, at each processing stage the software generates a quality report that serves to evaluate whether the relative accuracy of the project is good or not, comparing coincidences between 2D key points, vertices, and lines, which indicate how many points of union two or more images share.

The results obtained were transformed into digital cartography using the measurement tools available in a GIS environment (ArcMap and Globalmapper). From this information, the digital elevation model determined the direction of the water flow of the Chalan Gorge and certain details that are not observed from the surface. In addition, geological areas of interest for the field visit were identified to recognize the Geological Formations, classifying them according to their lithology. Likewise, satellite images from Google Earth Pro were used to identify important flaws, key morphologies for subsequent field verification ( Lanis & Razuvaev, 2018). From this preliminary information, the area of the Chalan Gorge and the area near the marmites were covered in 5 days to corroborate the information collected.

## Results and discussion

**Figure 1.**
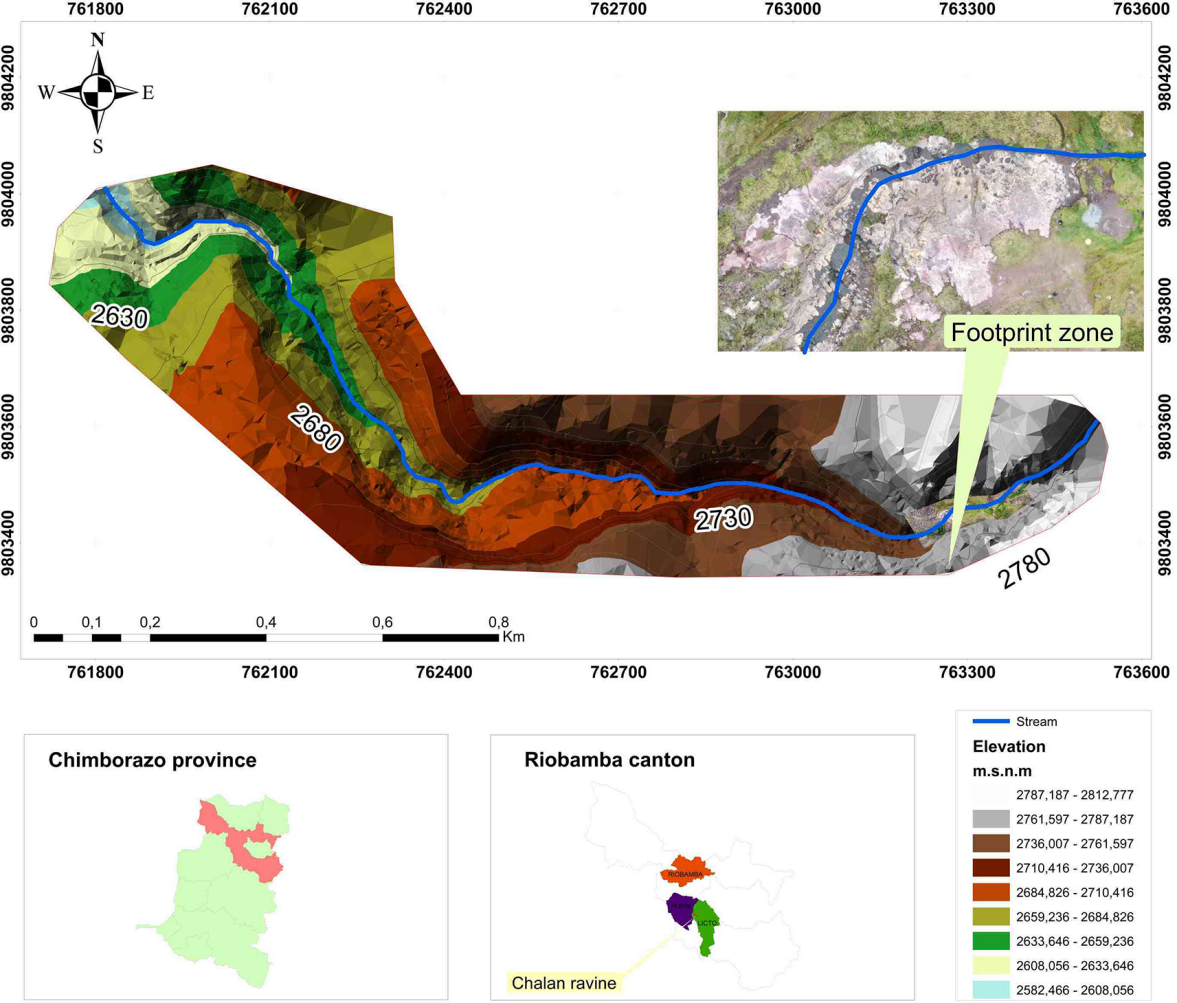
Location map of the Quebrada Chalán.

The Quebrada de Chalán ( Figure 1) is located in the south-central area of the Ecuadorian Inter-Andean Valley, specifically on the border of the Licto and Punín parishes, in Riobamba, Chimborazo province. It was formed from the slopes of the Tulabug volcano (3336 meters above sea level [masl]). The average altitude of the Chalán gorge is 2953 masl. (coordinates: 17M 763279.11/9802664), it is located 15 km from the city of Riobamba by the Riobamba-Macas road ( Román, 2010)

.

In the dry seasons (July to December) there are completely arid areas and other moistened spaces that provide a small but constant flow of water, that add up as it descends, thickening the flow of the stream. The land at the top of the ravine has little slope; in the middle part, this changes to a deep bed with slopes between rugged and steep, with steep flanks; in short sections the ravine slope decreases, causing the formation of sinks, holes and the water drainage by fractures in the rocks and the porosity of the soil. At the bottom, the ravine narrows to form a V, and the water current forms rapid and small jumps, until it flows into the Colorada ravine, named for its reddish strata ( Reinoso, 1974).

### Geological context

As described by Sauer (1965), Wolf (1892), Clapperton and Vera (1986) and Buenaño (2019) ( Buenaño, 2019), the Chalán ravine is located in the geological unit called the Cangahua Formation (Cangagua), this unit in the province of Chimborazo has a maximum power or thickness of 22 m. This Cangahua formation is the result of the volcanic activity of the Tulabug, which produced fine pyroclasts, easily transportable by the wind, that were deposited in the depressions of the inter-Andean valley, or in stagnant lakes; in certain areas they were consolidated, but without developing any stratification. Sauer (1965) determined that, according to the mineralogical composition of the andesites and dacites present in the formation, these originated after the second glaciation. In addition, what was described by Román (2010) determines that the Chalán ravine belongs to the Upper Pleistocene, specifically to the Third Interglacial Phase, since this is shown by the ichnofossil content ( *Coprinisphaera ecuadoriensis*) present in the Cangahua geological unit ( Sauer, 1965). The topsoil has variable thickness, composed of fine powder of whitish coloration with many cangagua balls; these fossil spheres serve as guide horizons to establish the relative age of the other strata, accumulated in thicknesses of several centimeters, as a result of the frequent volcanic eruptions in this area ( Reinoso, 1974).

### Paleontological context

The Chalán gorge is recognized for its paleontological and archaeological richness, as it describes fauna from the late Pleistocene and the Prehistory of man in Ecuador ( Román, 2010). In this context, as described by settlers, there are “bones of giants” in the surroundings of the ravine, being recorded for the first time by the chroniclers of the Indies in stories and legends, which alluded to ancient races of giants that would have populated these places in times immemorial. Juan de Velasco (1789) in his work “History of the Kingdom of Quito” describes the biological importance of the country and the presence of this type of gigantic bones buried in different strata of the soil and in several localities of the country. From these findings, those Ecuadorian legends of giants and strange beings that populated past times east were born ( Reinoso, 1974).

There are several groups of fossil mammals present in the Pleistocene fauna of the Chalán quebrada and its surroundings ( Wagner, 1883). Branco (1938) described in detail the ungulates of the area, especially equidae, camelids and cervids. As described by Román (2013) in 1894, the first mastodon discovered in the Pleistocene site of Quebrada Chalán was excavated. The remains of this specimen were preserved in good condition of fossilization at the Central University of Quito.

In this context, there are many notes from the national press that affirm the possibility that the marks on the rocks of the upper part of the ravine are traces of mastodons, the same that can be observed since a flood uncovered this area ( Maggi, 2016; Moncayo, 2019; Yurak, 2020; La Prensa, 2021).

### Prospecting in the Chalán ravine

On 8, 9 and 10 September 2021, several surveys were carried out through the ravine in order to obtain a detailed geological description of the area. Most of the outcrops were in inaccessible places, however, observations that could be made from a long distance revealed that the strata are sandwiched between white and yellow layers. The white strata are made up of fine grain given to the popcorn structure that covers these strata; on the other hand, the yellow strata are made up of a somewhat coarser grain that does not allow the formation of said structure. According to the works presented by Sauer (1965), these strata correspond to poorly consolidated volcanic tuffs.

**Figure 2.**
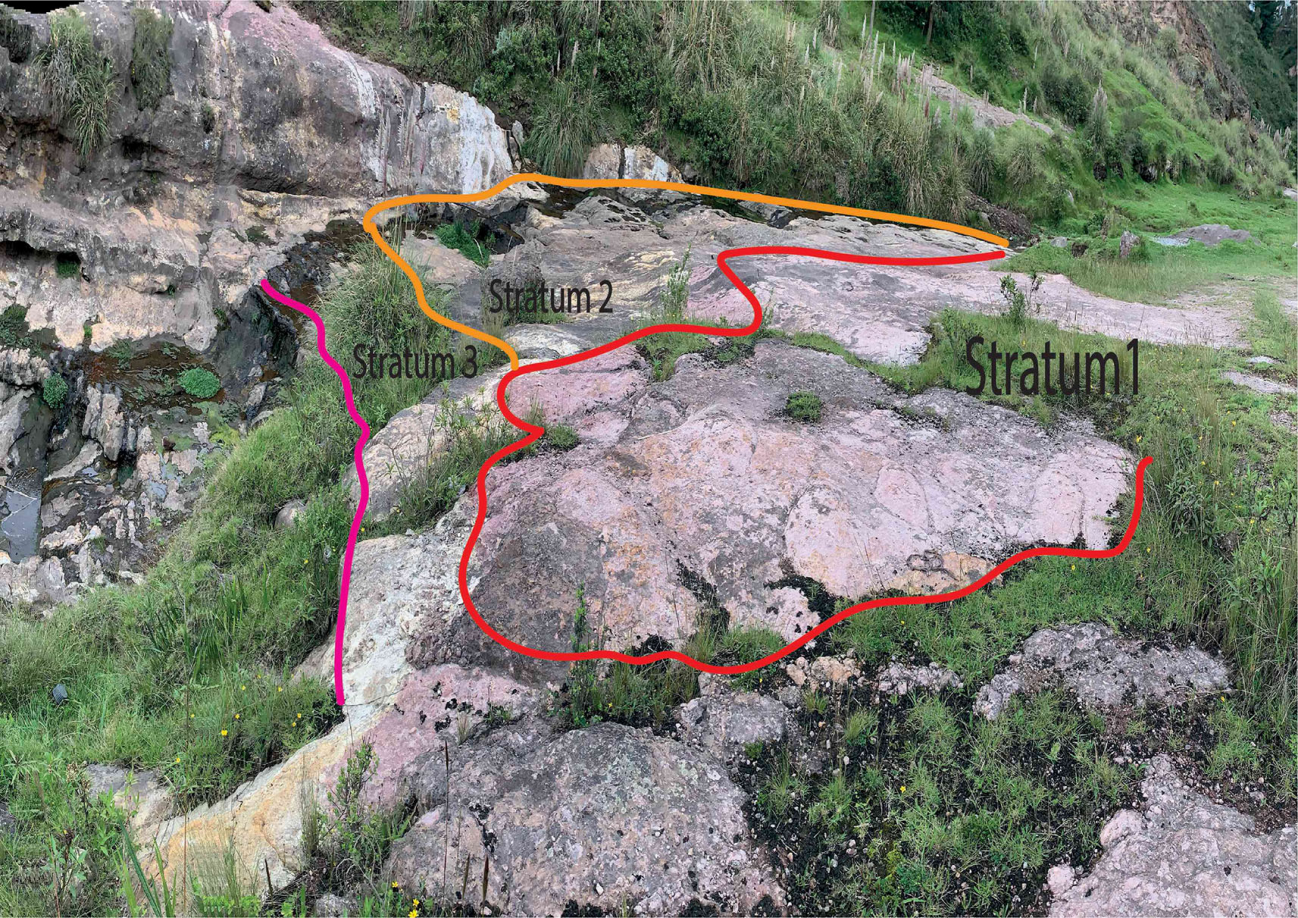
Stratigraphic succession of the study area.

The area of greatest interest of the Chalán revine is located at the coordinates 17M 763287,78 East/9803423,94 North at an altitude of 2963 masl, and corresponds to an outcrop formed by three strata arranged in the form of terraces resulting from water erosion. The terraces are the result of the different degrees of resistance to erosion that each stratum presents, as shown in Figure 2.

**Figure 3.**
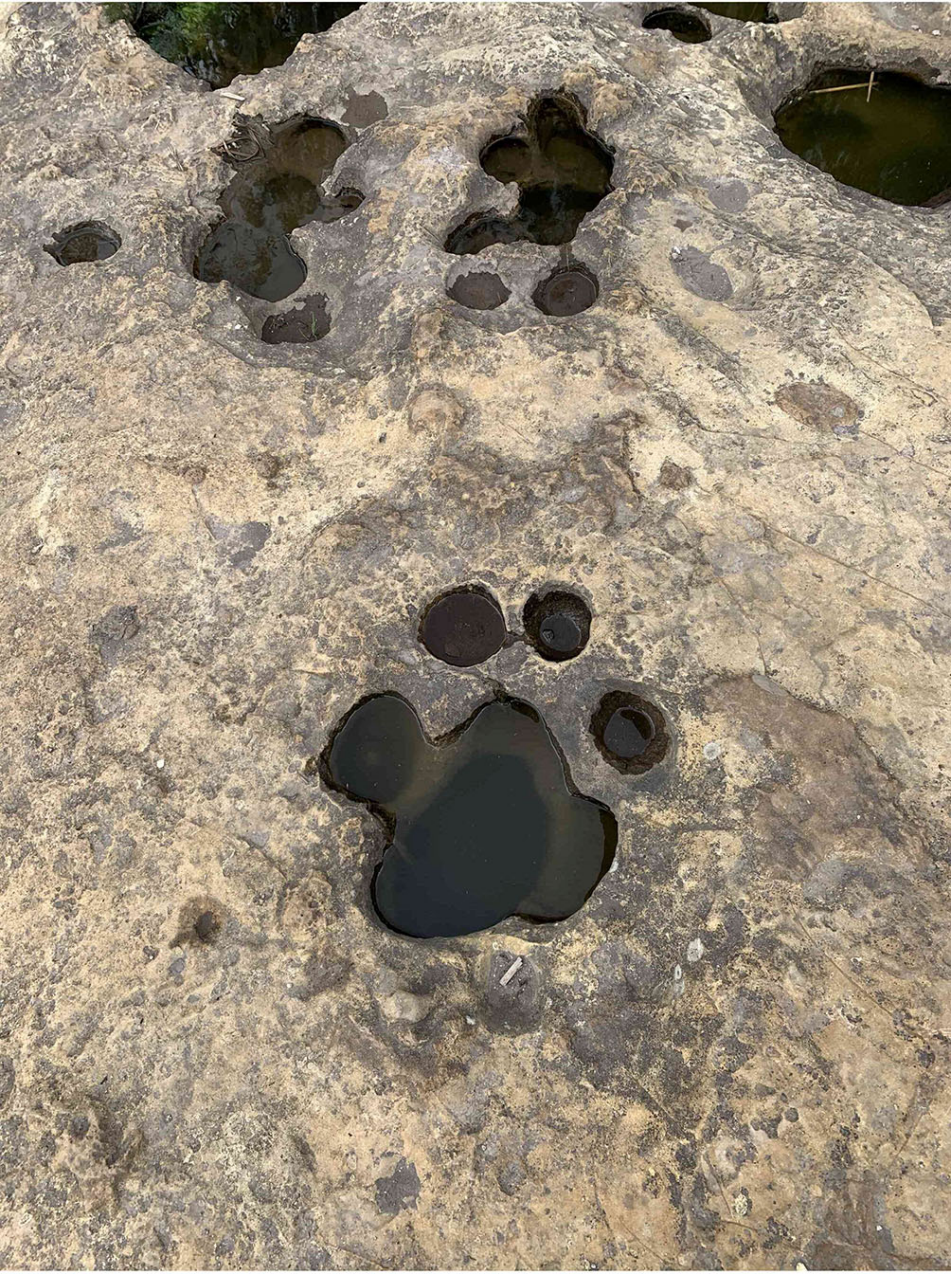
Circular markings located on the roof of stratum 2.

Stratum 1 was the most superficial and of which the thickness was not possible to measure, since its roof of this was eroded. It is reddish (possibly the result of weathering by water), it did not present stratifications, was well consolidated; the clasts inside were sub-regulatory, their sizes varied between 1 to 5 mm, poorly ordered, supported matrix, made up of fine grains; the mineral content corresponded mostly to plagioclase quartz and hornblendas. Stratum 2 bordered 1, by means of a concordant contact, had a thickness of 2 m, was yellowish, had no stratifications, was well consolidated; the clasts inside were angular, its size varied from 2 to 15 mm, without order, supported matrix, made up of fine grains, without mineral content. On the roof of the stratum there were a series of marks ranging from 8 to 30 cm in diameter ( Figure 3), due to their characteristics and according to Sauer (1965) this stratum was classified as a volcanic tova of chemical affinity towards an andesitic or dacite composition.

Stratum 3 limits with stratum 2 by a concordant contact; it is not possible to measure its thickness since the base of this is not seen; it was white, although in areas it was purple as a result of weathering by water; it did not present stratifications, was well consolidated, without clast content; it was made up of very fine grains without mineral content.

**Figure 4.**
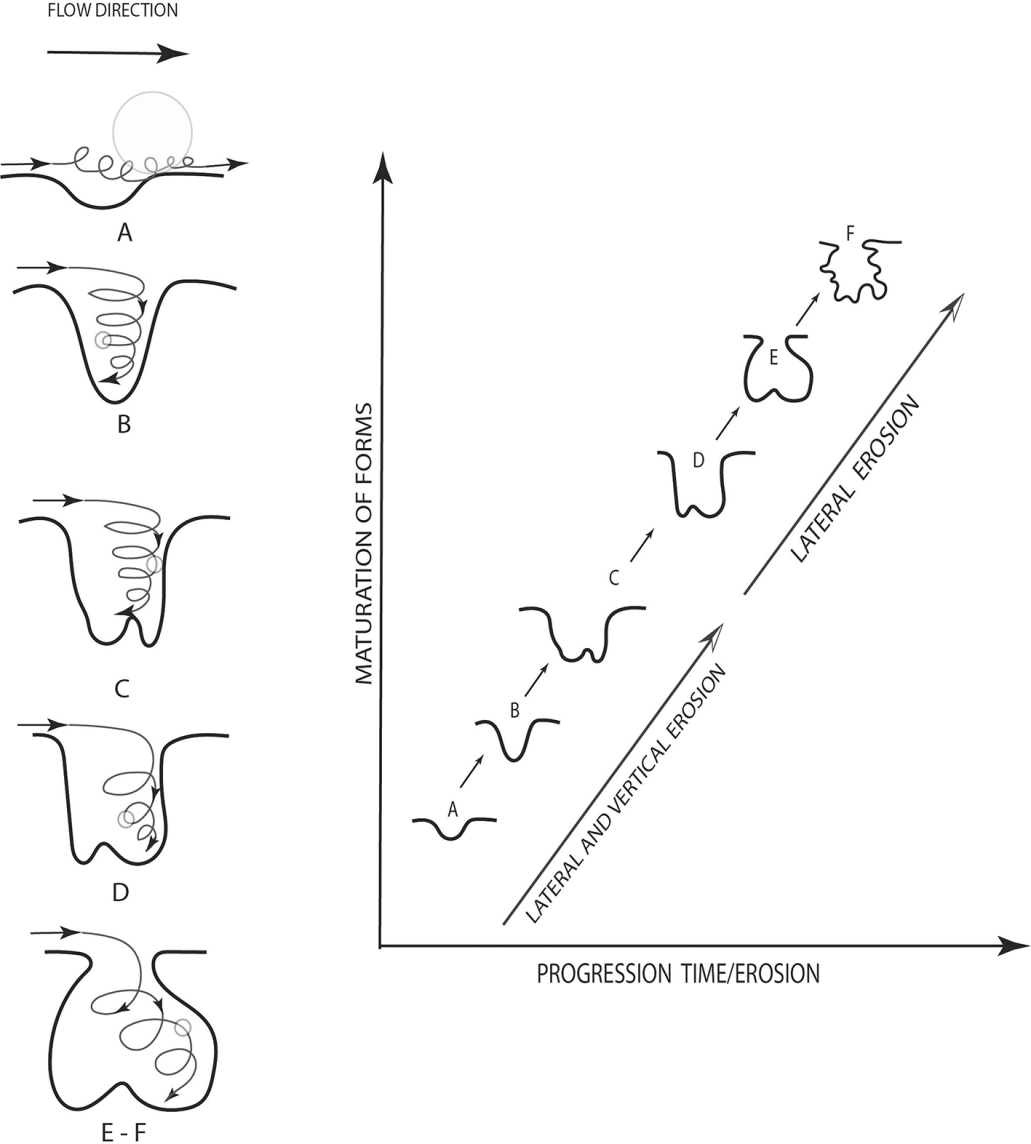
Description of the evolution of marmite erosion ( Lorenc, Muñoz, & Saavedra, 1995).

This type of erosion is known as giant marmites, the formation process of which is induced by defects in the bed producing flow alterations, generating turbulence or whirlpools. At this point the diaclases have an important role in the beginning and progress of the formation of the marmites ( Ortega, Gómez, Perez & Wohl, 2014; Pelletier, Sweeney, Roering and Finnegan, 2015). The way in which the marmites are formed is described in Figure 4, in which the evolution of erosion with respect to time is evidenced ( Lorenc, Muñoz, & Saavedra, 1995).

**Figure 5.**
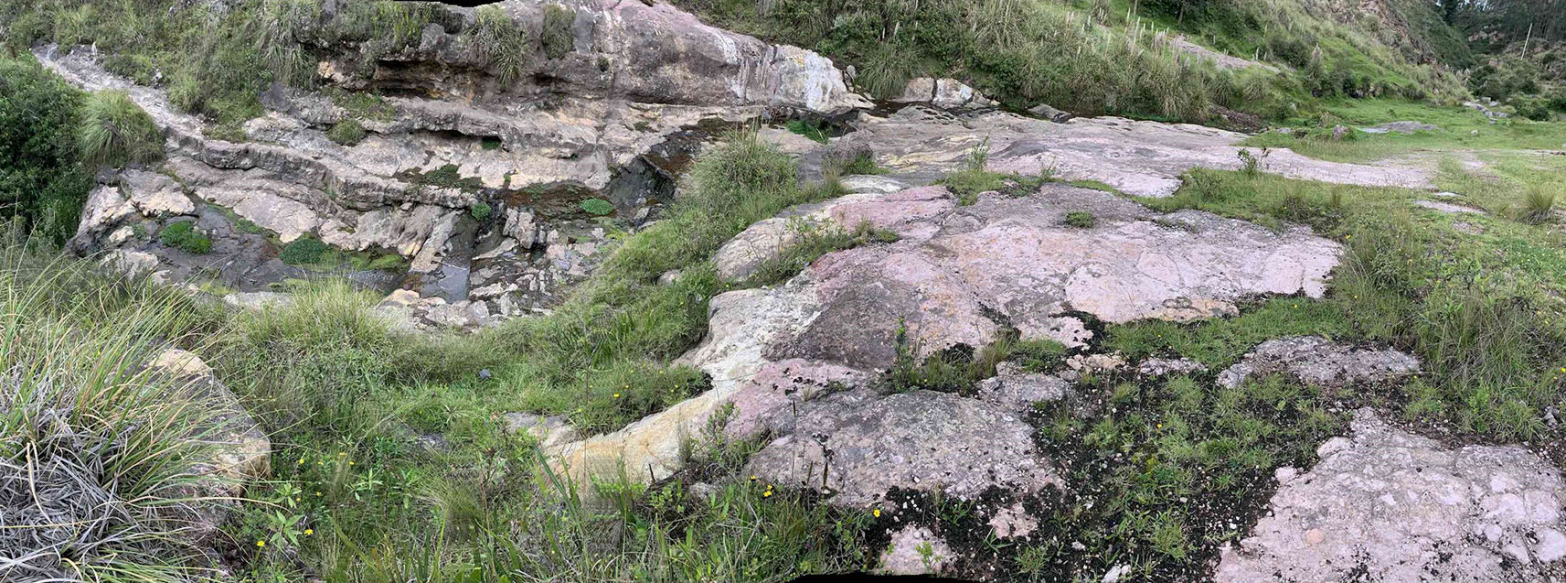
Water erosion in the upper part of the Chalán ravine.

Figure 5 shows in a general way the erosion that occurs in the upper part of the Chalán ravine. This is stuated just before a wall of approximately 10 m of altitude.

**Figure 6.**
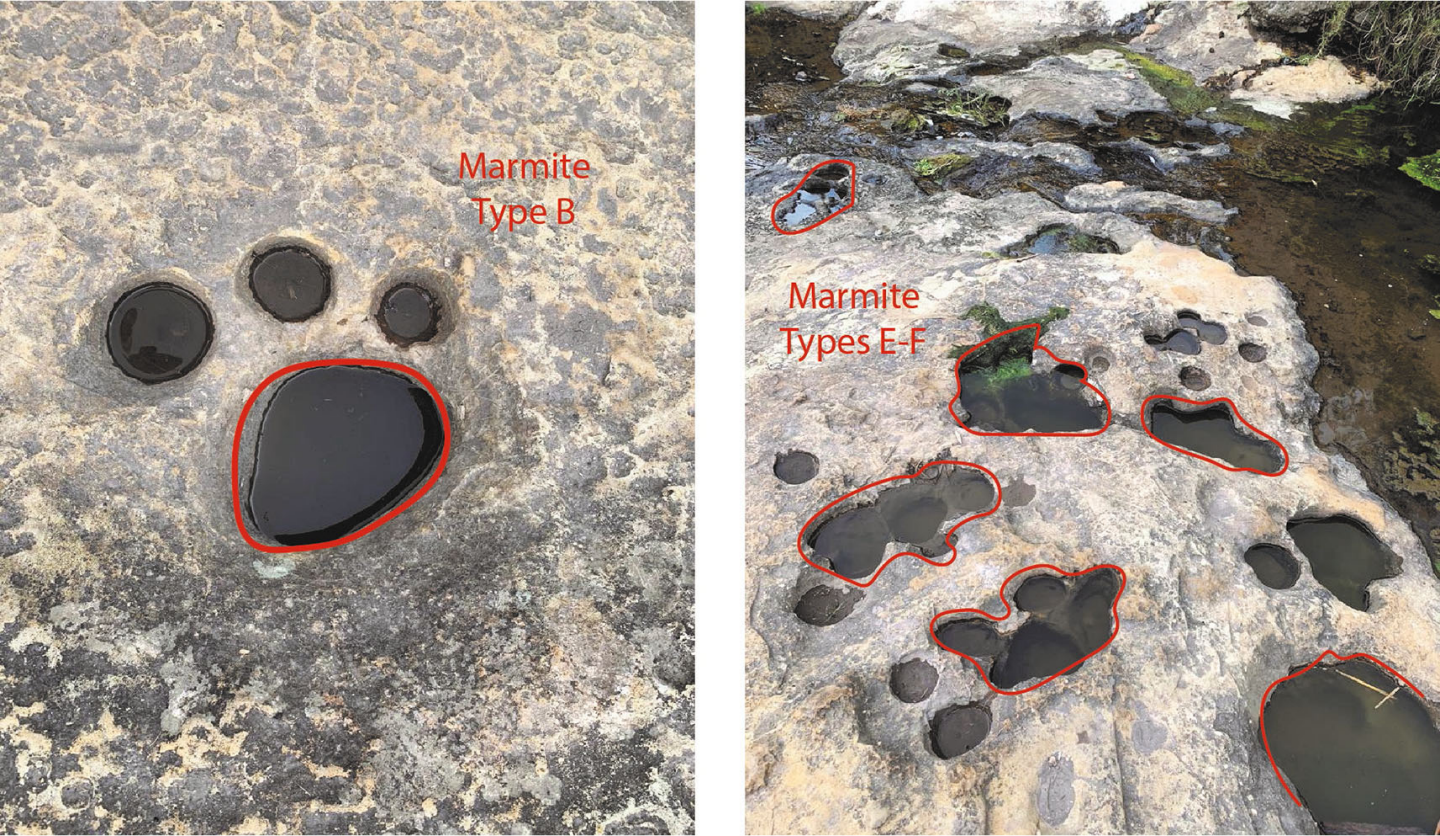
Marmites found in the upper part of the Chalán ravine.

The geological evidence and the erosion that shows the soil from the geomorphological point of view the formations that are in the upper part of the Chalán ravine correspond to marmites ( Figure 6). They had various diameters and depths. As it is necessary to categorise each of them, there were marmites of type A (erosion caused by natural abrasion less than 50 cm in diameter and depth), B, C, D (these three are deeper abrasions, where the particles cannot be lifted by vertical energy), E (in this type lateral erosion predominates, developing angular edges in the upper parts of the holes) and F (these are of the asymmetric type, favoring the tangential flow of the water, observing the formation of other marmites) as described by ( Lorenc, Muñoz, & Saavedra, 1995).

## Conclusions

Although it is true that the Chalán ravine has a large number of paleontological remains of Pleistocene fauna and megafauna, perhaps one of the most important in Andean territory so that even today the remains are visible with the naked eye in several walls of the lower areas of the Quebrada, the study area has known a significant erosion over time.

Precisely this erosion, produced above all by the water that passed through the ravine, is what has led to the form of the marmites that have been confused first, and mythologized later, as a series of mastodons fleeing before one of the eruptions of the Tulabug volcano.

The photogrammetric study, the arrangement of the marmites, their shape, the study of runoff and the geological interpretation, have allowed to determine that these features were not fossilized mastodon footprints, but erosion of the indicated strata wwhich, with the passage of time and the conditions described above, have caused this type of hole in the form of footprints in the rock.

## Data Availability

Zenodo: Mastodon footprints or water erosion in the Quebrada de Chalán (Licto, Ecuador), https://zenodo.org/record/6959979 ( Mendoza *et al., *2022). This project contains the following underlying data: CHALAN 2_dtm.prj (orthophoto obtained with the drone, the digital elevation model of the terrain) Data are available under the terms of the Creative Commons Attribution 4.0 International license (CC-BY 4.0).
