# Mastodon footprints found to be water erosion in the Quebrada de Chalán (Licto, Ecuador)

**DOI:** 10.12688/f1000research.123579.2

**Published:** 2023-07-25

**Authors:** Benito Mendoza, Mauro Jiménez, Pedro Pedro Carretero, Jhonnatan Hernández, Jennifer Loaiza, Daniela Brito, Geonatan Peñafiel

**Affiliations:** 1 Facultad de Ingeniería, Universidad Nacional de Chimborazo, Riobamba, Chimborazo, 060150, Ecuador; 2 Earth Sciences Energy and Environment, YACHAY, Urcuquí, Imbabura, 100650, Ecuador; 3 BMTLAB Laboratories and Engineering, Riobamba, Chimborazo, 060150, Ecuador

**Keywords:** Andean geology, mastodon footprints, water erosion, Chalán

## Abstract

The Chalan ravine is a deep bed creek that runs through Licto (Ecuador). It has been known since the 19th century for the abundance of paleontological remains of Pleiostocene fauna and megafauna in its profiles, where entire remains of mastodons were recovered. The abundance of these remains made one of the high areas, where marmites exist in different forms, was traditionally considered as mastodon footprints. Archaeological prospecting, geographic information system (GIS) technology, unmanned aerial vehicle (UAV), photogrammetry, and the geological study of the place, allowed us to determine that the mythical traces of mastodon were marmites made by the water erosion produced in the same ravine over time.

## Introduction

In the late 19th century, foreign researchers began to arrive in the Quebrada de Chalán, attracted by the legend of the existence of giant hominid bones. It was Juan Félix Proaño who, in 1884, after a major collapse in the Quebrada, proceeded to excavate the remains of a complete mastodon that had been exposed. These remains were sent to the Central University of Ecuador (Quito), where they disappeared after a large fire ( Branco, 1938; Román, 2010).

Later, other scholars such as Spillmann (1931) and Hoffstetter (1952) arrived, attracted by the abundance of Pleistocene animal remains, eager to conduct their research.

Therefore, the region is one of the main Pleistocene sites in Ecuador in terms of fossil remains from that period. With the arrival of these scholars in the first half of the 20th century, new legends emerged, one of which is the subject of our study, suggesting that the marmites area was actually fossilized mastodon footprints formed as the mastodons fled before the eruption of the nearby Tulabug volcano. This legend has been accepted as truth, being “endorsed by foreign scientists,” and even though it was never documented in a scientific study, it has been transmitted among the local inhabitants to this day. Consequently, even today, all the residents in the area believe that the marmites correspond to the aforementioned fossilized mastodon traces, leading to the creation of information panels and a marketing campaign in the area. Newspapers such as “La Prensa de Riobamba,” “Diario de Riobamba,” or “El Telégrafo” (national) still consider these features as mastodon traces and occasionally publish reports about the mentioned footprints, perpetuating the myth and confusing both locals and visitors ( La Prensa, 2021; de Riobamba, 2019; El Telegrafo, 2016).

The objective of this research was to determine whether the existing marks in the study area were the result, as tradition suggests, of mastodon tracks left by the mastodons that lived in the area during the Pleistocene, or if they were caused by water erosion (marmites) of the rock resulting from the passage of water.

## Methods

A visual archaeological surface survey was conducted in the study area, which determined that there are no archaeological or paleontological remains in the marmite area ( Fernández, 1989). For this purpose, aerial photogrammetry was utilized using an unmanned aerial vehicle (drone) equipped with a camera to obtain qualitative and quantitative information about the Earth’s surface through the recording, measurement, and interpretation of photographic images. The specific drone used for aerial digital cartography was a DJI Phantom 4 Pro V2.0 multirotor drone with a 20 MP camera designed for aerial photography. The generation of photogrammetric products involved three stages ( Casella, Drechsel, Winter, Benninghoff, & Rovere, 2020; Gasparini, Moreno-Escribano, & Monterroso-Checa, 2020; Stott, Williams, & Hoey, 2020):

• In the first stage, the flight path or flight plan was defined using Pix4DCapture software, configuring parameters such as the flight area in hectares, flight time in minutes, number of images to be captured, flight height in meters, pixel size in cm/px, recommended horizontal and longitudinal overlap of 75% as suggested by the software, speed in m/s, camera angle of 90°, and the number of batteries to be used.
• The second stage involved the preliminary survey of the terrain and the placement of ground control points (GCP) for accurate orthophoto georeferencing and desired elevation models.
• The third stage consisted of the actual flight, which took place on July 15, 2021, at an altitude of 150 meters, lasting 18 minutes and utilizing two batteries. A total of 199 aerial images were captured during the flight, with a spatial resolution of 4.5 cm/px.

The captured photographs were stored in the drone’s internal memory and then downloaded to the computer. The images were processed using the Pix4DMapper software, following the methodology recommended by the manufacturer and described below:

• When the software was launched, the option for creating a new project was selected, and the path where the postprocess files would be stored was defined.
• After generating the new project, the drone-captured photographs were added to the software. The software automatically detected the camera used and the coordinate system, which in this case were geographical coordinates. These coordinates were then transformed within the software to the Universal Transverse Mercator (UTM) coordinate system.
• For 3D processing, the option for 3D Maps processing was selected to generate the orthomosaic, point cloud, and digital elevation models. During the initial processing, the image scale and geometrically verified pairing were determined.
• The dense point cloud was processed with classification to improve the generation of the digital terrain model (DTM), digital surface model (DSM), and orthomosaic. Additionally, an orthorectification and contour generation process was performed with a 5 m interval.

In addition, at each processing stage, the software generates a quality report that evaluates the relative accuracy of the project by comparing the coincidences between 2D key points, vertices, and lines. These measurements indicate the number of shared points between two or more images, providing an assessment of the project’s accuracy.

The obtained results were then transformed into digital cartography using the measurement tools available in a Geographic Information System (GIS) environment, specifically ArcMap ( ESRI, 2020) and Global Mapper ( Blue Marble Geographics, 2021). The digital elevation model derived from the data allowed for determining the water flow direction in the Chalan Gorge and capturing certain details that are not observable from the surface. Furthermore, geological areas of interest were identified based on the digital cartography, facilitating subsequent field visits to recognize geological formations and classify them according to their lithology. Additionally, satellite images from Google Earth Pro were utilized to identify significant features and key morphologies that required further verification during fieldwork ( Lanis & Razuvaev, 2018). Based on this initial information, a comprehensive field investigation was conducted over a period of five days to verify and validate the data obtained. The investigation focused on the Quebrada de Chalán area and the vicinity of the marmites.

## Results and discussion

**Figure 1.**
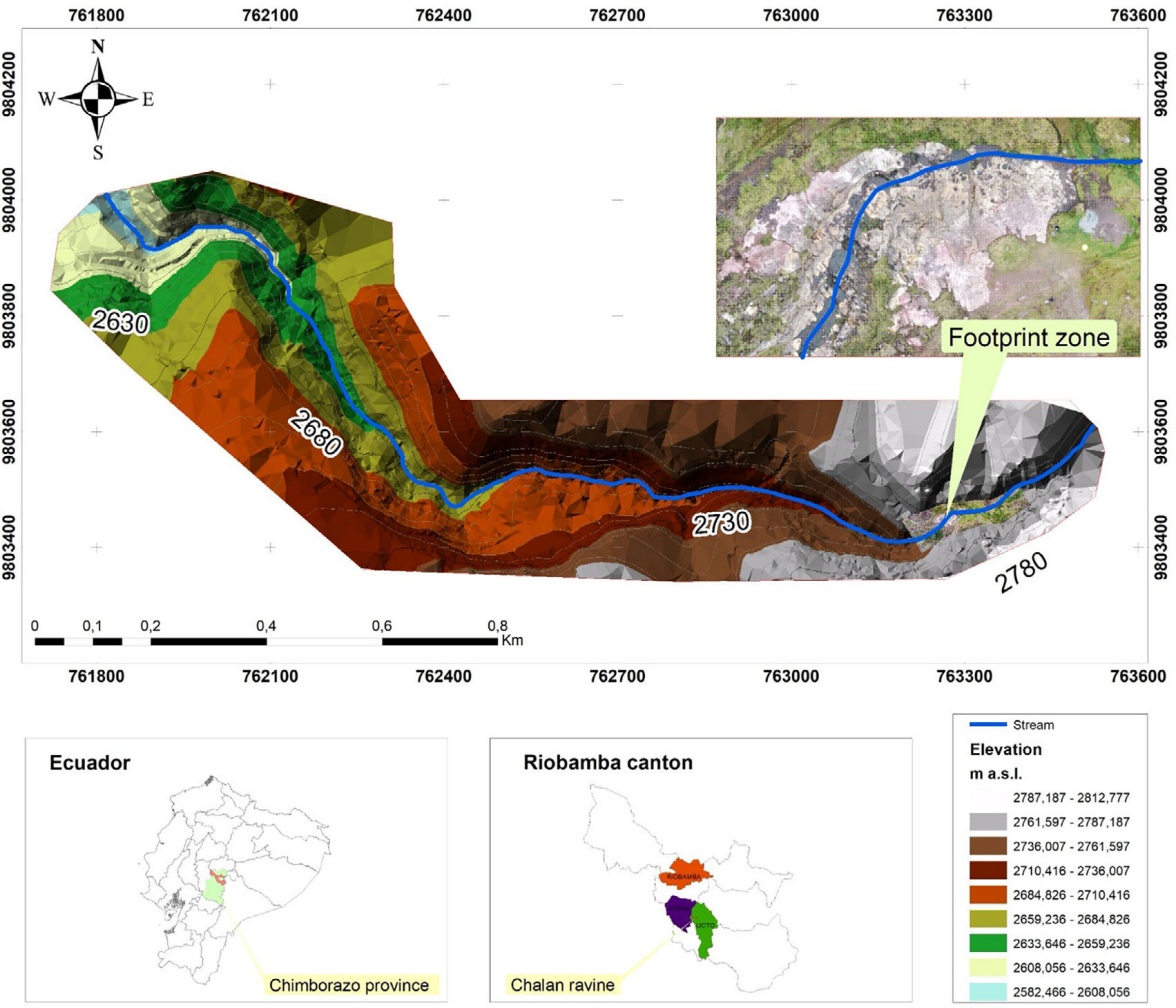
Location map of the Quebrada Chalán.

The Quebrada de Chalán ( Figure 1) is situated in the south-central region of the Ecuadorian Inter-Andean Valley, specifically on the border between the Licto and Punín parishes, in Riobamba, Chimborazo province. It was carved out by the slopes of the Tulabug volcano, which stands at an elevation of 3336 meters above sea level (m a.s.l.). The average altitude of the Chalán Gorge is 2953 m a.s.l. Its coordinates are 17M 763279.11/9802664. The gorge is located approximately 15 km from the city of Riobamba, accessible via the Riobamba-Macas road ( Román, 2010).

In the dry seasons (July to December), there are completely arid areas and other moistened spaces that provide a small but constant flow of water, contributing to the stream’s volume as it descends and leading to an increase in the water flow. The terrain at the top of the ravine has a gentle slope, while in the middle part, it transforms into a deep bed with rugged and steep slopes, characterized by steep flanks. In certain sections, the ravine’s slope decreases, resulting in the formation of sinks, holes, and water drainage through fractures in the rocks and soil porosity. Towards the bottom, the ravine narrows to take on a V-shape, and the water current creates rapids and small waterfalls before eventually merging with the Quebrada Colorada, named for the reddish hue of its strata ( Reinoso, 1974).

### Geological context

As described by Sauer (1965), Wolf (1892), Clapperton and Vera (1986) and Buenaño (2019) ( Buenaño, 2019), the Quebrada de Chalán is located in the geological unit called the Cangahua Formation (Cangagua). This formation, in the province of Chimborazo, has a maximum thickness of 22 m. The Cangahua Formation is the result of the volcanic activity of the Tulabug, which produced fine pyroclasts easily transportable by the wind. These pyroclasts were deposited in depressions of the inter-Andean valley or in stagnant lakes, and in certain areas, they were consolidated without developing any stratification. Sauer (1965) determined that the andesites and dacites present in the formation originated after the second glaciation, based on their mineralogical composition. Additionally Román (2010) described that the Chalán ravine belongs to the Upper Pleistocene, specifically to the Third Interglacial Phase. This determination is supported by the presence of ichnofossils ( *Coprinisphaera ecuadoriensis*) found in the Cangahua geological unit ( Sauer, 1965). The topsoil in the area has variable thickness and is composed of fine powder with a whitish coloration and many cangagua balls. These fossil spheres serve as guide horizons to establish the relative age of other strata, which accumulated in thicknesses of several centimeters as a result of frequent volcanic eruptions in the region ( Reinoso, 1974).

### Paleontological context

The Quebrada de Chalán is recognized for its paleontological and archaeological richness, as it contains fauna from the late Pleistocene and evidence of prehistoric human presence in Ecuador ( Román, 2010). Within this context, local settlers have described the presence of “bones of giants” in the vicinity of the ravine, which were first recorded by chroniclers of the Indies in stories and legends. These accounts alluded to ancient races of giants that supposedly inhabited the region in times immemorial. Velasco (1789) in his work “History of the Kingdom of Quito” described the biological importance of the country and mentioned the discovery of gigantic bones buried in different strata of the soil and various locations across the country. From such findings, Ecuadorian legends about giants and strange beings from the distant past emerged ( Reinoso, 1974).

The Pleistocene fauna in the Quebrada de Chalán and its surroundings contains several groups of fossil mammals ( Wagner, 1883). Branco (1938) provided a detailed description of the ungulates in the area, particularly equids, camelids, and cervids. As described by Román (2013), the first mastodon discovery in the Pleistocene site of Quebrada de Chalán was excavated in 1894. The remains of this specimen were well-preserved as fossils and are currently housed at the Central University of Quito.

In this context, numerous reports from the national press support the possibility that the marks on the rocks in the upper part of the ravine are mastodon footprints. These marks became visible after intense rainfall events caused significant runoff and soil erosion. Even today, as the soil continues to erode, more of these indentations in the rocky bed keep appearing ( Maggi, 2016; Moncayo, 2019; Yurak, 2020; La Prensa, 2021).

### Prospecting in the Quebrada de Chalán

On 8, 9 and 10 September 2021, several surveys were conducted through the ravine to obtain a detailed geological description of the area. Most of the outcrops were in inaccessible places, however, observations made from a long distance revealed that the strata are interleaved between white and yellow layers. The white strata consist of fine grains giving rise to popcorn structure covering these layers; on the other hand, the yellow strata are composed of slightly coarser grains that do not allow the formation of such a structure. Based on the works presented by Sauer (1965), these strata correspond to loosely consolidated volcanic tuffs.

**Figure 2.**
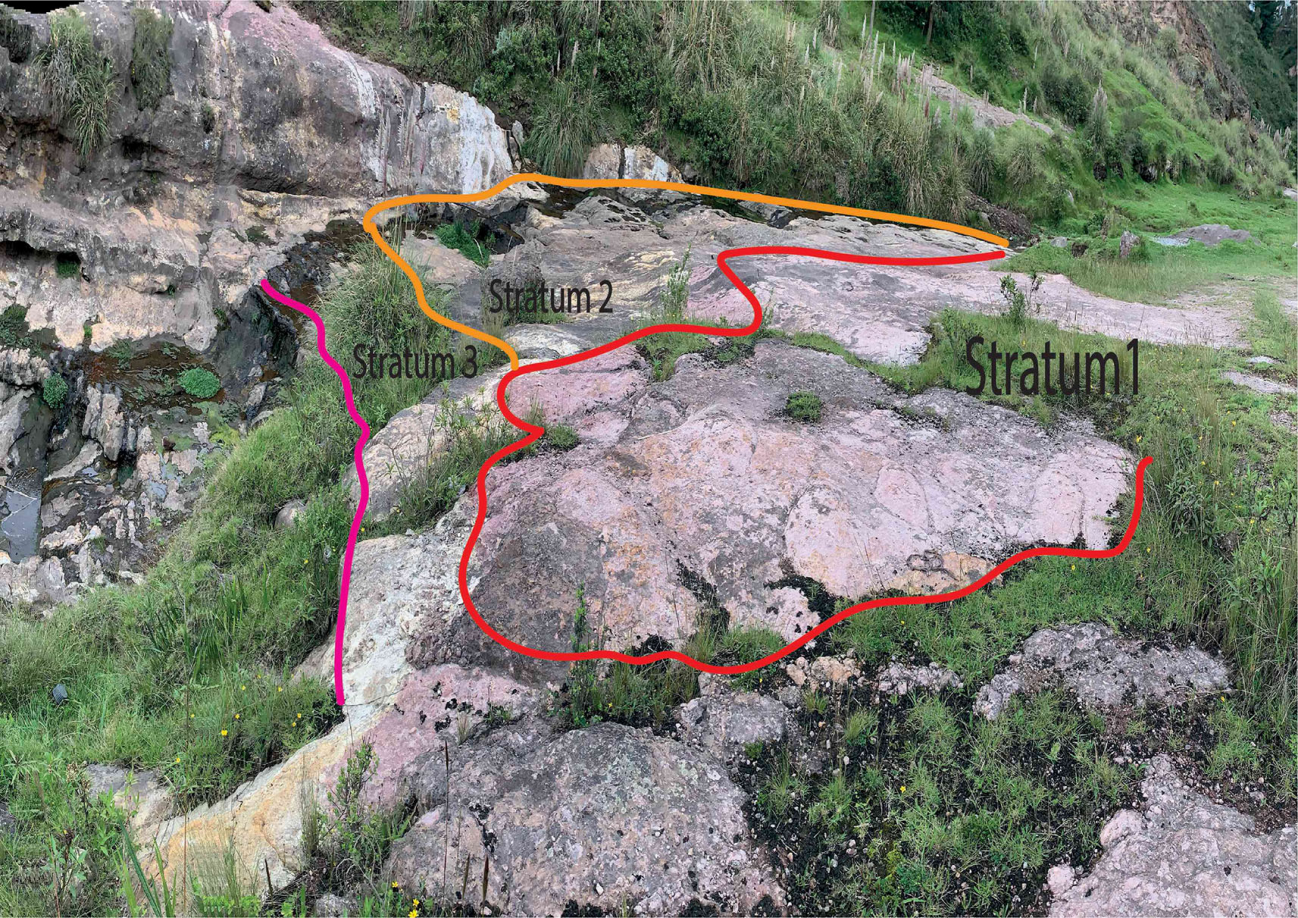
Stratigraphic succession of the study area.

The area of greatest interest of the Quebrada de Chalán is located at the coordinates 17M 763287,78 East/9803423,94 North at an altitude of 2963 m a.s.l. This location corresponds to an outcrop formed by three strata arranged in the form of terraces resulting from water erosion. The terraces are the result of the varying degrees of resistance to erosion exhibited by each stratum, as shown in Figure 2.

**Figure 3.**
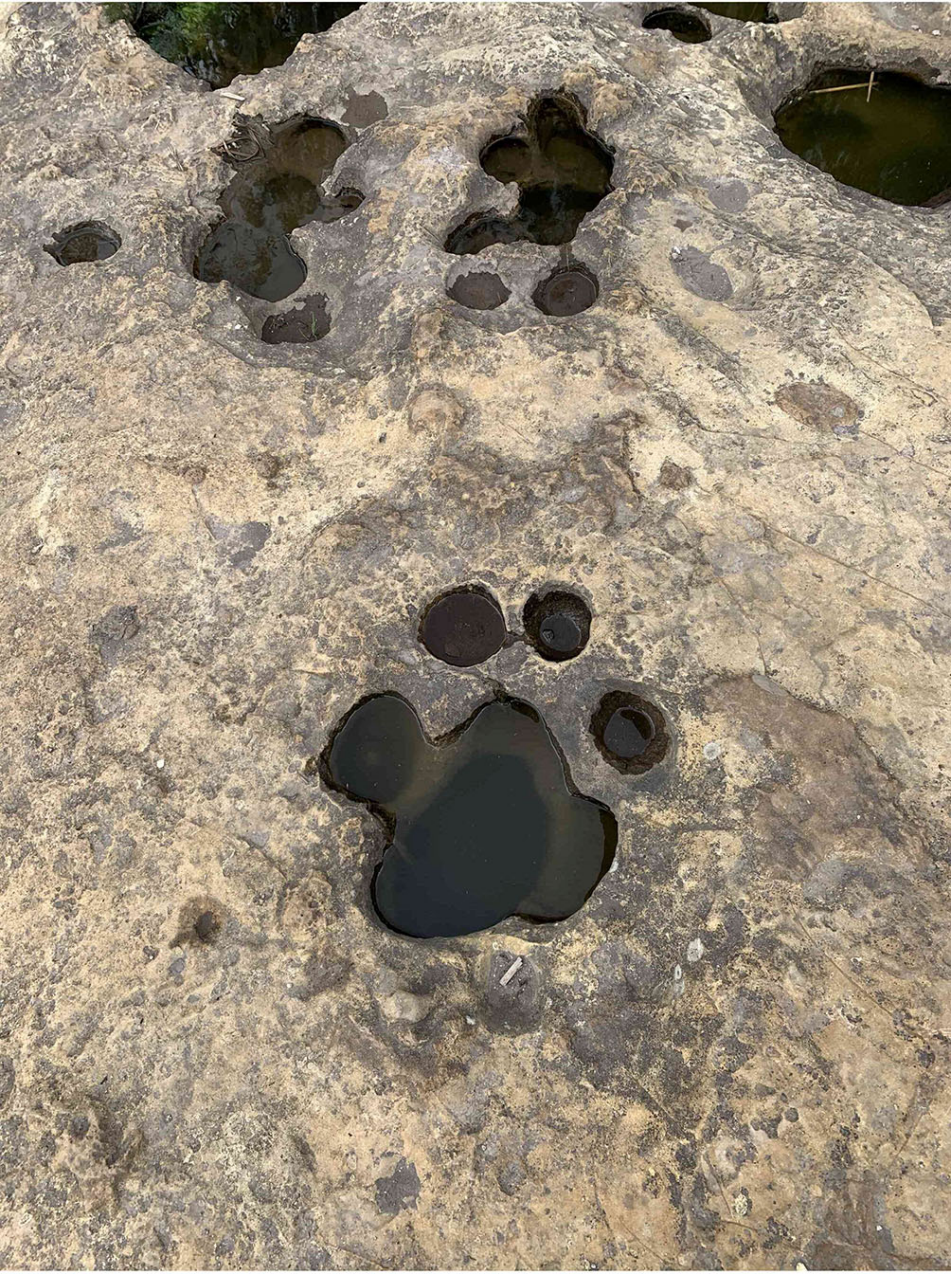
Circular markings located on the roof of stratum 2.

Stratum 1 was the most superficial, and its thickness could not be measured as its roof was eroded. It exhibited a reddish color, possibly the result of weathering by water, and lacked stratifications. The stratum was well-consolidated, and the clasts inside were sub-angular, varying in size between 1 to 5 mm, and poorly ordered. The matrix was composed of fine grains, primarily consisting of plagioclase quartz and hornblende minerals. Stratum 2, adjacent to Stratum 1 with a concordant contact, had a thickness of 2 m and appeared yellowish. It also lacked stratifications and was well-consolidated. Inside, the clasts were angular, ranging in size from 2 to 15 mm and without any specific order. The matrix was composed of fine grains without significant mineral content. On the roof of this stratum, there were a series of marks ranging from 8 to 30 cm in diameter ( Figure 3). According to its characteristics and in accordance with Sauer (1965) this stratum was classified as a volcanic tuff with a chemical affinity towards an andesitic or dacite composition.

Stratum 3 is bounded by stratum 2 through a concordant contact. Its thickness cannot be measured as the base is not visible. The stratum appeared white, although in certain areas, it exhibited a purple hue due to water-induced weathering. Similar to the previous strata, it lacked stratifications, was well-consolidated, and contained no clasts. The matrix was composed of very fine grains without significant mineral content.

**Figure 4.**
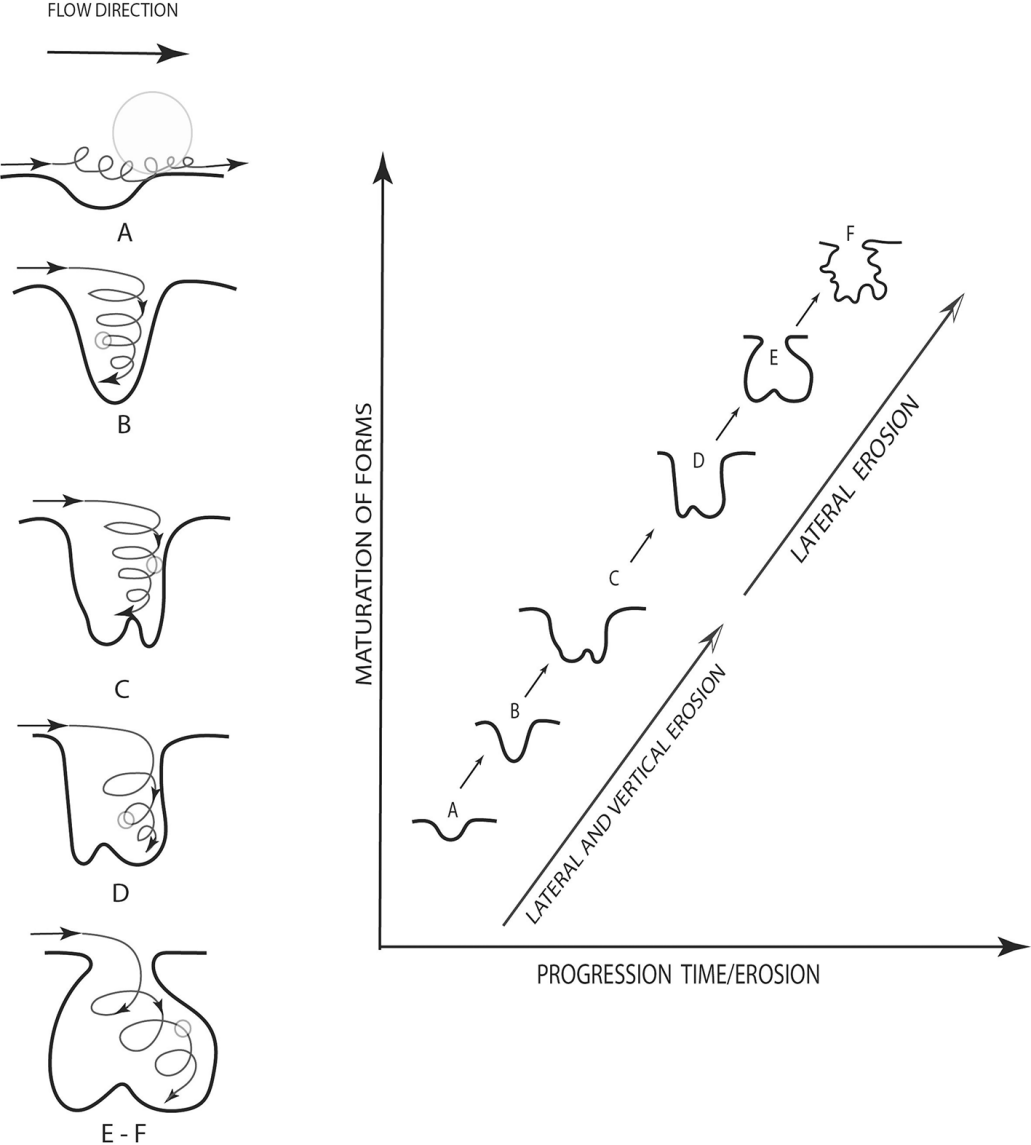
Description of the evolution of marmite erosion ( Lorenc, Muñoz, & Saavedra, 1995).

To understand the erosive process, that stream potholes are erosive morphologies associated with fluvial processes such as abrasion or hydraulic erosion. These develop in diverse types of substrates, from soft materials like clays to resistant bedrock such as granites. They are also present in fluvial channels on rocky beds with varying levels of incision. Additionally, the erosion process is induced by defects in the bed that generate flow alterations, producing turbulence or whirlpools. At this point, joints play an important role in the initiation and progression of the formation of the potholes, and they are related to the position and geometric configuration of the channel. Similarly, lithology plays a significant role in the erosion of rivers in rocky beds, affecting it at different scales, from valley geometry to sculpted forms. Potholes can form from minor depressions resulting from weathering or the impact of large rocks ( Ortega, Gómez, Perez & Wohl, 2014; Pelletier, Sweeney, Roering & Finnegan, 2015; Kale, & Joshi, 2004). A schematic of how potholes are formed is described in Figure 4, which highlights the evolution of erosion over time ( Lorenc, Muñoz, & Saavedra, 1995).

**Figure 5.**
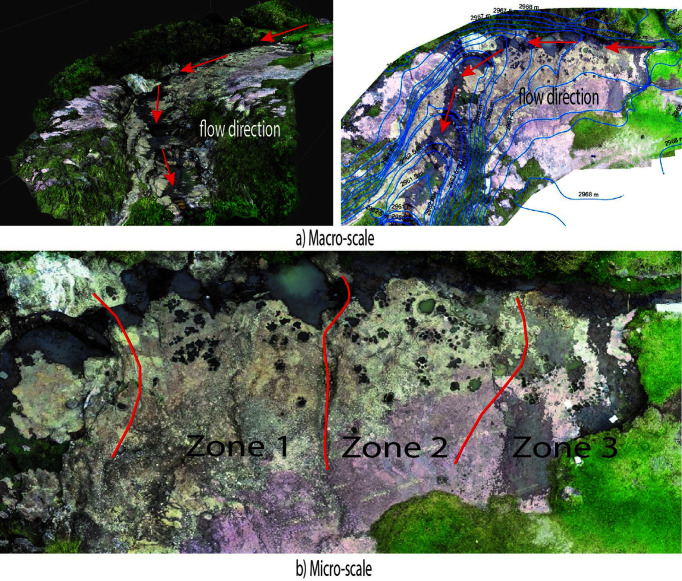
a. Macro scale, flow direction in the upper part of the Quebrada de Chalán. b. Micro scale zones of identification of holes.

Following the methodology used by Ortega *et al.* (2014), a macro-scale analysis of the area where the mastodon footprints (potholes) are located was conducted. It was observed that the water flow originates from 2780 to 2730 meters above sea level (m a.s.l.), where the formation of a waterfall is evident ( Figure 5a). In the micro-scale analysis, the number of holes was counted in three zones: Zone 1 with 36 holes, Zone 2 with 140 holes, and Zone 3 with 18 holes. The characteristics of the holes are heterogeneous, with diameters ranging from 10 cm to 70 cm ( Figure 5b).

Similarly, to understand the origin of the footprints, it is necessary to know that the predominant mastodon species in Latin America was Cuvieronius hyodon, which ranged across the Andes from Ecuador to Chile ( Mothè *et al.*, 2016). The diet of South American mastodons has been extensively studied in recent years through dental enamel microwear analysis and oxygen and carbon isotope studies ( Dantas *et al.*, 2022; Dominato *et al.*, 2010). However, there are only two known records of mastodon footprints in South America, both of which are found in Chile, specifically in Punta Pelluco in the Lakes Region, this area is renowned for its fossil forest from the late glacial period (Late Pleistocene) and also includes tracks of horses and camelids from the late Pleistocene deposits of Pehuén-Co and Laguna del Monte in the Buenos Aires Province ( Oliva & Arregui, 2018). However, there are only two known records of mastodon footprints in South America. One set of footprints is found in Chile, specifically in Punta Pelluco, in the Lakes Region. This area is renowned for its fossil forest from the late glacial period (Late Pleistocene), and the footprints found there also include traces of camelids ( Campos-Medina *et al.*, 2022). Similarly, the records obtained in Argentina come from late Pleistocene deposits of Pehuén-Co and Laguna del Monte in the Buenos Aires Province, and they also include tracks of horses and camelids. These footprints appear to have been left by a medium-sized proboscidean, allowing the identification of pathologies that affect the legs of modern elephants (and ungulates) in this individual ( Oliva & Arregui, 2018).

**Figure 6.**
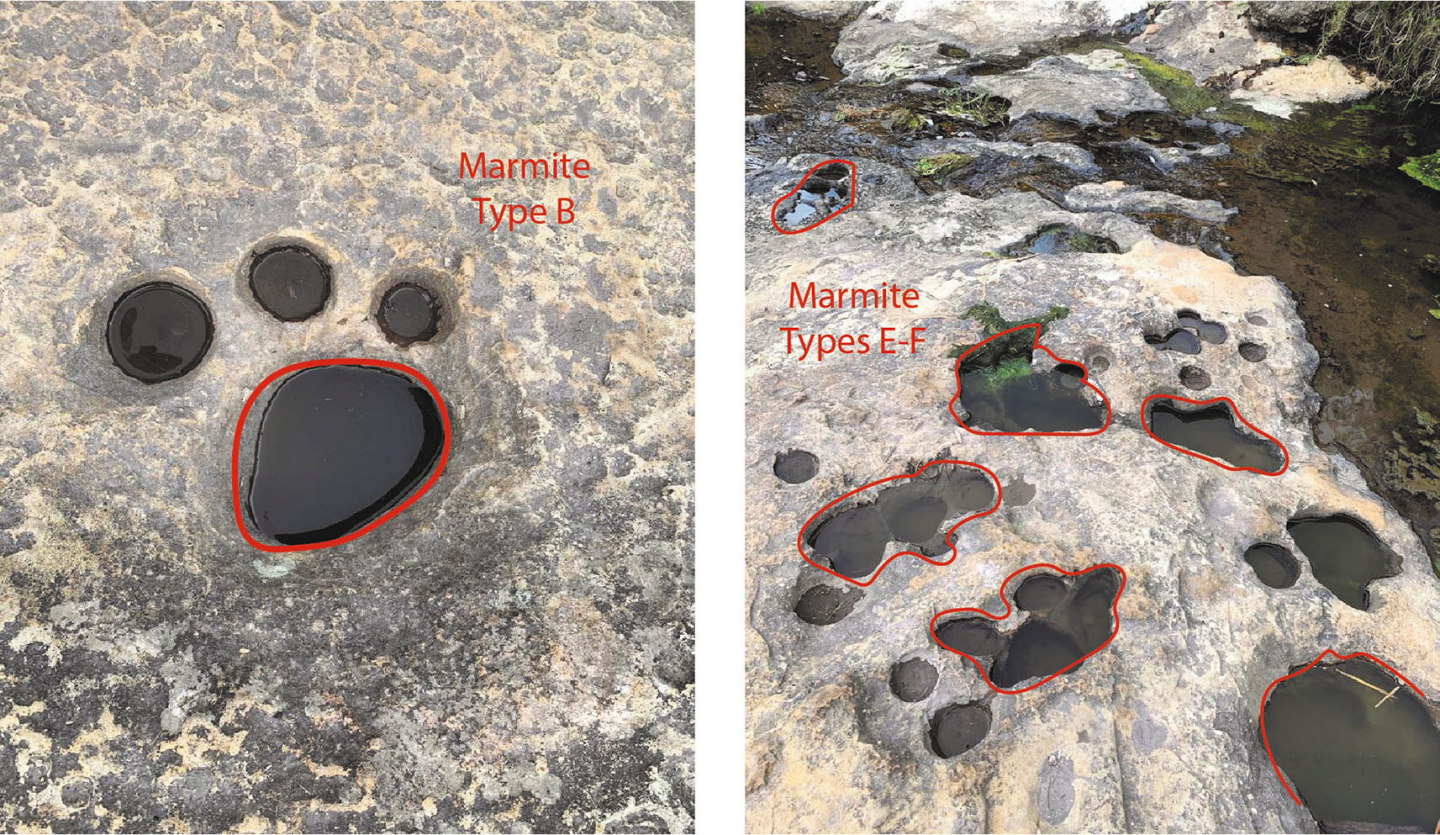
Marmites found in the upper part of the Chalán ravine.

In this context, having geological evidence, microscale arrangement, and the forms of erosion found in the upper part of the Chalán Ravine, the shapes of these holes correspond to geological formations known as “marmites” ( Figure 6) from a geomorphological perspective. These marmites exhibit different diameters and depths, so they have been classified as follows: Type A, characterized by natural abrasion with diameters and depths less than 50 cm; Types B, C, and D, which are deeper abrasions where particles cannot be lifted by vertical energy; Type E, where lateral erosion predominates, leading to the development of angular edges at the top of the holes; and Type F, which are asymmetric and favor tangential water flow, often resulting in the formation of other marmites. These categorizations were described by Lorenc, Muñoz, and Saavedra (1995).

## Conclusions

Although it is true that the Quebrada de Chalán has a large number of paleontological remains of Pleistocene fauna and megafauna, perhaps one of the most important in the Andean territory, where even today remains can be seen on several walls in the lower areas of the Quebrada, the upper part of the ravine, where this study was conducted, shows significant erosion over time.

Precisely this erosion, primarily caused by wall collapses in the ravine and water running through it, gives rise to the features that were initially mistaken and later mythologized as a series of mastodon footprints fleeing before one of the eruptions of the Tulabug volcano. However, these features are actually potholes. This conclusion was reached through photogrammetric study, macro and micro-scale analyses, arrangement of the potholes, their shape, water flow analysis, and geological interpretation. These analyses revealed that these features are not fossilized mastodon footprints but rather erosion of the rock layers, which, over time and under the described conditions, create these hole-like formations in the rock.

To fully complete the study and demonstrate that they are potholes, future analysis of the dimensions of the formations using geophysical techniques such as the Schmidt hammer test is necessary. Additionally, comparing these results with the footprints found in archaeological sites in Chile and Argentina would further strengthen the evidence supporting their classification as potholes.

## Data availability

### Underlying data

Zenodo: Mastodon footprints or water erosion in the Quebrada de Chalán (Licto, Ecuador), https://zenodo.org/record/6959979 ( Mendoza *et al.,* 2022).

This project contains the following underlying data:

CHALAN 2_dtm.prj (orthophoto obtained with the drone, the digital elevation model of the terrain)

Data are available under the terms of the Creative Commons Attribution 4.0 International license (CC-BY 4.0).
