# Supplementary material for: Mastodon footprints found to be water erosion in the Quebrada de Chalán (Licto, Ecuador)
Source: F1000Res. 2023 Jul 25;11:1239. Originally published 2022 Nov 1. [Version 2] doi: 10.12688/f1000research.123579.2 (PMC10442588; doi:10.12688/f1000research.123579.2)
Supplement: Peer review history

# Peer review: Reviewer response for version 2

**DOI:** 10.5256/f1000research.153115.r196115

**Published:** 2023-08-21

**Authors:** Adrienne Mayor

**Affiliations:** 1 Classics Department and the History and Philosophy of Science Program, Stanford University, Stanford, California, USA

Since the nineteenth century, paleontologists have excavated numerous well-preserved Pleistocene mastodon remains in the abundant fossil deposits around Quebrada de Chalán, a deep ravine in south-central Ecuador. The abundance of mastodon fossils led to the assumption that a series of round holes in the bedrock above the deep-bed stream were footprints left by mastodons. For more than 100 years, this identification became embedded in traditional descriptions of the Chalán Gorge’s geological treasures. Only two other locales in South America, Chile and Argentina, boast mastodon tracks reported in 2018 and 2022, respectively.

 The co-authors thoroughly analyzed the geophysical and hydrological data of the Chalán Gorge and the properties of the holes and their context. Their interpretation of the evidence concluded that the “mastodon tracks” are really marmites created by water erosion in the ravine over millennia. There are clear descriptions and images showing how turbulent fluvial action carve the rounded holes in the streambed over time. I find the article well-conceived and organized, with convincing conclusions.

 The authors might wish to expand the discussion of local and Indigenous legends about giant bones in Ecuador. This is mentioned only briefly. As early as the 1500s, Spanish explorers, such as Pedro Cieza de Leon, preserved accounts by Inca, Manta, Arica, and other Indigenous peoples about the bones of fearsome giants that invaded Ecuador and were destroyed en masse, by fire. (see A. Mayor, F *ossil Legends of the First Americans, *Princeton 2005, pp 80-84). The giants’ bones were actually the fossil remains of mastodons and other megafauna of the Pleistocene, extremely plentiful in southern Ecuador.

 Also, given that marmites figured in a modern explanatory myth perpetuated by scientists for more than a century, the authors might consider looking for previous local explanations of the pot holes often called “Giants’ kettles” or “cauldrons” in other cultures, to see whether Indigenous inhabitants of the region, not just Europeans, tried to account for the remarkable formations along the gorge.

Is the work clearly and accurately presented and does it cite the current literature?

Yes

If applicable, is the statistical analysis and its interpretation appropriate?

Not applicable

Are all the source data underlying the results available to ensure full reproducibility?

Yes

Is the study design appropriate and is the work technically sound?

Yes

Are the conclusions drawn adequately supported by the results?

Yes

Are sufficient details of methods and analysis provided to allow replication by others?

Yes

Reviewer Expertise:

Fossil folklore, history of science, legends and myths about fossilization

I confirm that I have read this submission and believe that I have an appropriate level of expertise to confirm that it is of an acceptable scientific standard.

## References

[1] : Fossil Legends of the First Americans. *Princeton* .2005;80-84

# Peer review: Reviewer response for version 2

**DOI:** 10.5256/f1000research.153115.r190452

**Published:** 2023-08-01

**Authors:** Valeria Lupiano

**Affiliations:** 1 National Research Council of Italy, Research Institute for Geo-Hydrological Protection, CNR-IRPI, Perugia, Italy

I believe the article has reached a sufficient level of improvements and is ready for indexing.

Is the work clearly and accurately presented and does it cite the current literature?

Yes

If applicable, is the statistical analysis and its interpretation appropriate?

Not applicable

Are all the source data underlying the results available to ensure full reproducibility?

No source data required

Is the study design appropriate and is the work technically sound?

Yes

Are the conclusions drawn adequately supported by the results?

Partly

Are sufficient details of methods and analysis provided to allow replication by others?

Partly

Reviewer Expertise:

Geology, geomorphology, landslides

I confirm that I have read this submission and believe that I have an appropriate level of expertise to confirm that it is of an acceptable scientific standard.

# Peer review: Reviewer response for version 1

**DOI:** 10.5256/f1000research.135699.r163369

**Published:** 2023-03-14

**Authors:** Matthew Bennett

**Affiliations:** 1 Institute for Studies in Landscapes and Human Evolution, Bournemouth University, Poole, UK

This is an interesting paper which documents erosional scours previously thought to be mastodon footprints. While I agree with their conclusions, they do not describe the traces in sufficient detail to ensure that others can benefit from their work. More detailed description of the traces is really needed, close up scans or photogrammetry models. Some morphometric comparison with known proscibean footprints would strengthen the work. The evolutionary model for the scours does not seem supported by any data I can see. It may well be accurate but it needs to be evidenced. Work in progress is my assessment and the authors should be encouraged to revise the article.

Is the work clearly and accurately presented and does it cite the current literature?

Partly

If applicable, is the statistical analysis and its interpretation appropriate?

Not applicable

Are all the source data underlying the results available to ensure full reproducibility?

No source data required

Is the study design appropriate and is the work technically sound?

Partly

Are the conclusions drawn adequately supported by the results?

Partly

Are sufficient details of methods and analysis provided to allow replication by others?

Partly

Reviewer Expertise:

Footprints

I confirm that I have read this submission and believe that I have an appropriate level of expertise to state that I do not consider it to be of an acceptable scientific standard, for reasons outlined above.

# Response

**Published:** 2023-07-17

**Authors:** Benito Mendoza

**Affiliations:** Universidad Nacional de Chimborazo, Ecuador

Dear reviewer, while the presented work requires more data to determine whether the traces are mastodon footprints or erosional scours, a thorough revision has been conducted. Additional paragraphs have been added to reinforce the initial theory presented in the paper, supported by a literature review and macro and microscale analysis. It is important to note that there are limited indications of mastodon footprints in South America, and this topic is just beginning to be studied. Consequently, the conclusions leave open the possibility of furthering this research through geophysical methods and comparative analysis with similar features found in Argentina and Chile. This will be explored in future work to ascertain whether these are indeed mastodon footprints or erosional scours.

# Peer review: Reviewer response for version 1

**DOI:** 10.5256/f1000research.135699.r159655

**Published:** 2023-01-12

**Authors:** Valeria Lupiano

**Affiliations:** 1 National Research Council of Italy, Research Institute for Geo-Hydrological Protection, CNR-IRPI, Perugia, Italy

The authors present a paper that aims to show how the footprints, found in Quebrada de Chalán (Licto, Ecuador), attributed to Mastodon by local people, are instead a geomorphological phenomenon (river potholes) related to fluvial erosion and the lithology of the site. They did a field survey and used aerial photogrammetry to prove this.

 The manuscript is well structured but needs a revision of English language.

 It is necessary to explain the mechanism that leads to the formation of river potholes by commenting on Figure 5. Also mention the factors that predispose to the phenomenon. See for example:

1. Ortega, J. A., Gómez-Heras, M., Perez-López, R., & Wohl, E. (2014). Multiscale structural and lithologic controls in the development of stream potholes on granite bedrock rivers. Geomorphology, 204, 588-598.
2. Kale, V. S., & Joshi, V. U. (2004). Evidence of formation of potholes in bedrock on human timescale: Indrayani river, Pune district, Maharashtra. Current Science, 723-726.

The conclusions are very meager, should be supplemented with some consideration.

 Minor revision:

1. figure 1: insert a small map with Ecuador location; In the legend change "m.s.n.m." in "m a.s.l.";
2. In the text change "masl"  in "m a.s.l.";
3. ArcMap and globalmapper cite as:
  ESRI year. ArcMap, Release xx. Redlands, CA: Environmental Systems Research Institute.
  Blue Marble year. Globalmapper  Release....;

Is the work clearly and accurately presented and does it cite the current literature?

Yes

If applicable, is the statistical analysis and its interpretation appropriate?

Not applicable

Are all the source data underlying the results available to ensure full reproducibility?

No source data required

Is the study design appropriate and is the work technically sound?

Yes

Are the conclusions drawn adequately supported by the results?

Partly

Are sufficient details of methods and analysis provided to allow replication by others?

Partly

Reviewer Expertise:

Geology, geomorphology, landslides

I confirm that I have read this submission and believe that I have an appropriate level of expertise to confirm that it is of an acceptable scientific standard, however I have significant reservations, as outlined above.

## References

[1] : Multiscale structural and lithologic controls in the development of stream potholes on granite bedrock rivers. *Geomorphology* .2014;204: 10.1016/j.geomorph.2013.09.005 588-598 10.1016/j.geomorph.2013.09.005

[2] : Evidence of formation of potholes in bedrock on human timescale: Indrayani river, Pune district, Maharashtra. *Current Science* .2004;723-726

# Response

**Published:** 2023-07-17

**Authors:** Benito Mendoza

**Affiliations:** Universidad Nacional de Chimborazo, Ecuador

Dear reviewer, a thorough review of the English writing was conducted, taking into account minor corrections. Additionally, the study was analyzed with respect to new suggested literature, which led to further explanation on how the marmites could have originated.
